# Orthogeriatric care—outcome of different fragility fractures

**DOI:** 10.1007/s00402-023-04993-w

**Published:** 2023-07-22

**Authors:** Carlos Pankratz, Annika Risch, Jacob Oxen, Raffael Cintean, Alexander Boehringer, Florian Gebhard, Konrad Schuetze

**Affiliations:** Department of Trauma-, Hand-, Plastic- and Reconstructive Surgery, University Ulm Medical Centre, Albert-Einstein-Allee 23, 89081 Ulm, Germany

**Keywords:** Orthogeriatric care, Fragility fracture, Geriatric trauma, Surgical outcome

## Abstract

**Introduction:**

Fragility fractures (FF) are associated with increased morbidity and mortality and reflect a dramatic turning point in the life of older adults. The scientific discourse is dominated by proximal femoral fractures, but FF affect multiple parts of the body and often precede hip fractures. Orthogeriatric co-management has multiple shown to improve patient’s outcome. We hypothesize that all geriatric patients with FF benefit from orthogeriatric co-management.

**Materials and methods:**

We retrospectively evaluated all patients over 70 years with FF (hip joint, periprosthetic, spine, pelvic ring, and humerus) of our geriatric trauma center for the years 2019–2021, who received orthogeriatric co-management. Demographic data, fracture type, complications, discharge modality and in-hospital mortality were recorded. For patients transferred to geriatrics, the Barthel Index (BI) and the discharge modality were recorded. Primary outcome parameters were discharge modality and BI difference. Secondary outcome parameters were complication rates and in-hospital mortality. Logistic regression analysis was performed.

**Results:**

555 patients (83.8 ± 6.5 years, 182 males, 373 females) were evaluated. 245 (44.1%) patients were referred to geriatrics for further orthogeriatric treatment. Positive predictors were age, surgery, and a high Charlson Comorbidity Index. The overall in-hospital mortality was 8.6% (n = 48) (5.8% (n = 32) during acute trauma care and 6.5% (n = 16) during stay in geriatrics). The mortality rate of nursing home residents was significantly higher compared to patients living at home (10.4% vs. 5.6%). The rate of non-surgical complications was 44.5%. 26.9% of patients living at home were discharged to a nursing home, while 51.3% were able to return home. The risk of admission to a nursing home was reduced for thoracolumbar fractures (OR = 0.22) and increased markedly for periprosthetic fractures (OR = 3.95). During orthogeriatric treatment, all fractures showed a significant increase in BI. Patients living at home benefited more than nursing home residents (20.5 ± 19.5 vs. 8.7 ± 18.0 points). The chance of a BI increase (> 19 points) was increased for hip and pelvic ring fractures. Devastating results showed patients with dementia. In comparison, mentally healthy patients had a 4.5-fold increased chance of increasing their BI (> 19 points).

**Conclusions:**

Presented data shows that all patients with FF are at high risk for complications and could benefit from standardized orthogeriatric management. Modern patient care requires a holistic orthogeriatric approach to improve patient’s outcome.

## Introduction

Fragility fractures, caused by low-energy trauma, are associated with increased morbidity and mortality and often represent a dramatic turning point in the life of older adults [1–3]. Beside their serious impact on individual’s fate, fragility fractures becoming a major burden for healthcare systems and demographic change is expected to further increase fracture-associated costs in the next decades [4].

Geriatric patients are a vulnerable patient group, as they often suffer from multiple comorbidities and functional impairments. Associated with pre-existing limited physical and mental resources the geriatric patient is highly at risk for post-operative complications and increased mortality [5, 6], making treatment of those patients challenging.

Considering above-described developments and the special issues of the geriatric patient the liaison of orthopaedic surgeons and geriatricians in terms of multidisciplinary orthogeriatric care is still an emerging topic [7]. Albeit, different orthogeriatric care models are coexisting [6] an interdisciplinary approach in treating geriatric hip fracture patients has shown to increase postoperative clinical as well as cost-associated outcome measures in terms of length of stay, in-hospital mortality, long-term mortality and postoperative complications [8–10].

Hip fractures clearly dominating the scientific discourse [11], but fragility fractures occur in multiple parts of the human body, such as humerus, pelvis, spine or wrist [12] and often precede devastating hip fractures [13]. Data on orthogeriatric care in the course of different fragility fractures is poor [14]. Here, we hypothesize that not only hip fracture patient benefit from orthogeriatric care. For this purpose, we retrospectively evaluated the patient’s outcome of different fracture types in our orthogeriatric care of a level 1 trauma centre.

## Materials and methods

All patients ≥ 70 years with hip, proximal femur, periprosthetic, spinal, pelvic, and humerus fractures treated in our level 1 trauma center are routinely screened using the Identification of Seniors at risk (ISAR) score at first day of admission. All patient with ISAR score 2 or higher receive further standardized geriatric assessment and treatment by a geriatric consultant during hospitalization in our traumatology department until discharge. This complex early geriatric rehabilitative treatment includes regular interdisciplinary meetings, development of rehabilitation goals with focus on geriatric syndromes. Patient discharge is managed by social workers, attending surgeons and geriatric consultants. Patients, who discharge from acute trauma center to geriatrics, receive continuously weekly visits by an experienced trauma surgeon. The orthogeriatric care includes occupational therapy, physiotherapy, nutrition counseling, sport therapy and constant supervision by geriatrician and orthopedic trauma surgeons.

**Fig. 1:**
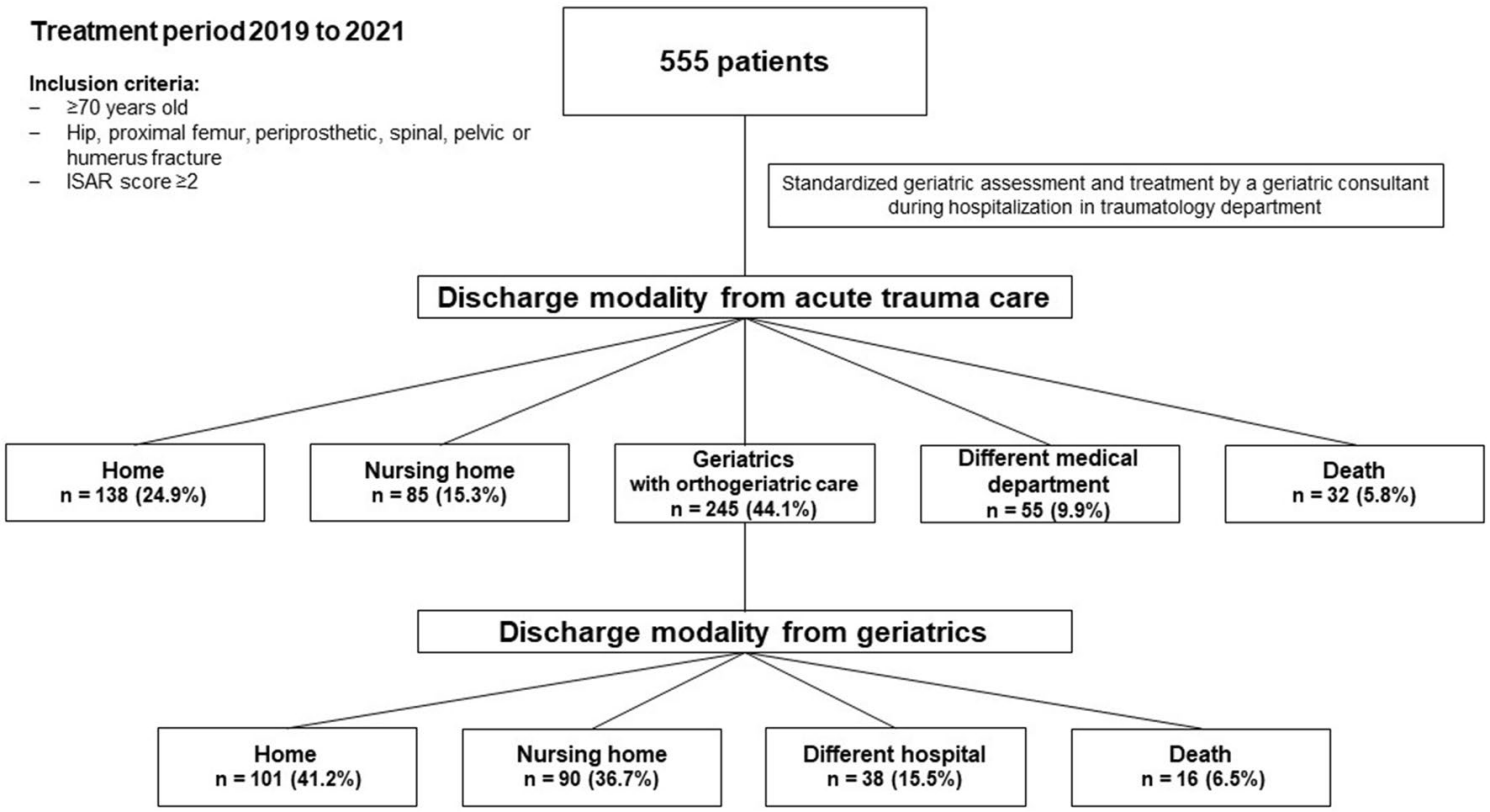
Study flow chart: a retrospective chart review. ISAR score: Identification of Seniors at risk score

For this study, we retrospectively reviewed all patients of our trauma department in the treatment period from 2019 to 2021 who received geriatric assessment and treatment (Fig. 1). Presented data were obtained with institutional and local ethical committee approval for the use of the data. All patient charts were reviewed for demographical data, type of injury, surgical and non-surgical complications, discharge destination and hospital mortality. In Patients discharged to geriatrics after trauma center Barthel index (BI) and home accommodation before and after treatment was recorded. Primary outcome measures were discharging modality and change in BI during orthogeriatric care. Secondary outcome measures were rate of surgical and non-surgical complications, and hospital mortality. Surgical complications like hematoma, bleeding, surgical site infection, implant failure or fracture displacement were recorded if surgical revision was needed. We recorded major non-surgical complications, like serious cardiovascular (e.g., thrombosis, embolism, infarction, stroke), infectious (pneumonia, urinary tract infection) events as well as acute organ failure (kidney, heart, and liver failure) and anemia requiring red blood cell transfusion.

Data analysis was performed using SPSS (v25.0, Co., IBM, Armonk, NY, USA). Demographic characteristics are described as mean and standard deviation, and categorical data as absolute and relative frequencies. For the primary and secondary categorical outcome measures logistic/ordinal regression was performed considering related variables to prevent confounding. A p-value < 0.05 was considered as statistically significant.

## Results

### Patient population

For 555 patients, medical records were reviewed. Out of these 555 patients, 182 were male and 373 were female. The youngest patient was 70, while the oldest was 104 years old. The mean age was 83.8 ± 6.5 years with a mean age adjusted Charlson Comorbidity Index (CCI) of 6. There were 128 patients with hip fractures, 158 with proximal femur fractures, 30 with periprosthetic fractures of the proximal femur, 41 with proximal humerus fractures, 93 with pelvic ring fractures, 74 with thoracolumbar fractures and 31 with cervical fractures. Overall, 87.4% (n = 485) of the fractures were treated surgically. Further data and complication rate shows Table 1. Table 2. shows the composition of non-surgical complications.

**Table 1:** General data

Fracture side	Age [y] ± SD	TTS [d] ± SD	LOS [d] ± SD	CCI [y] ± SD	Surgical treatment [n (%)]	In-patient mortality [n (%)]	Non-surgical complication rate [n (%)]	Surgical complication rate [n (%)]	Cases [n]
Hip	83.5 ± 6.4	0.5 ± 0.7	10.1 ± 4.5	5.7 ± 1.8	128 (100%)	5 (3.9%)	42 (32.8%)	6 (4.7%)	128
Proximal femur	85.9 ± 6.8	0.3 ± 0.6	9.7 ± 5.3	6.2 ± 1.8	151 (95.6%)	13 (8.2%)	81 (51.3%)	4 (2.5%)	158
Periprosthetic proximal femur	84.9 ± 6.7	1.7 ± 2.7	13.3 ± 11.8	6.0 ± 1.6	25 (83.3%)	1 (3.3%)	13 (43.3%)	1 (3.3%)	30
Proximal humerus	81.6 ± 7.1	2.3 ± 2.1	8.3 ± 4.2	5.4 ± 2.1	34 (82.9%)	1 (2.4%)	11 (26.8%)	5 (12.2%)	41
Pelvic ring	83.2 ± 5.8	4.2 ± 2.6	12.1 ± 6.3	5.6 ± 1.5	73 (78.5%)	4 (4.3%)	37 (39.8%)	1 (1.1%)	93
Thoracolumbar	82.5 ± 5.5	4.6 ± 2.5	11.8 ± 5.5	5.6 ± 1.9	55 (74.3%)	4 (5.4%)	26 (35.1%)	0 (-)	74
Cervical	82.0 ± 6.5	3.7 ± 2.5	7.7 ± 4.8	5.2 ± 2.0	18 (58.1%)	4 (12.9%)	9 (29.0%)	0 (-)	31

*TTS* time to surgery, *LOS* length of hospital stay, *CCI* age adjusted Charlson Comorbidity Index, *SD* standard deviation

**Table 2:** Non-surgical complications

Non-surgical complication type [n]	Cardiovascular - infarction - thrombosis - embolism - stroke [n/N (%)]	Infection - pneumonia - urinary tract [n/N (%)]	Organ failure - kidney - heart - liver - delirium [n/N (%)]	Red blood cell transfusion [n/N (%)]
346	65/555 (11.7%)	172/555 (31.0%)	38/555 (6.8%)	71/555 (12.8%)

### Discharge from acute trauma care.

Overall, 24.9% (n = 138) of the patients were discharged home, 44.1% (n = 245) into further orthogeriatric care (geriatrics with orthopedic consultant service), 15.3% (n = 85) into a nursing home, 9.9% (n = 55) into another medical department and 5.8% (n = 32) died during acute trauma care. For further analysis logistic regression was performed excluding cases with discharge modalities to another medical department, unknown destination, or death. Patient with high age, surgical treatment, non-surgical complications or high CCI were statistically more often admitted into orthogeriatric care (p < 0.05). Cervical fractures had a significantly higher chance of been released directly home compared to all other (p < 0.05). Discharge modality out of acute trauma care shows Tab. 3.

**Table 3:** Discharge modality for each fracture type after acute trauma care

Fracture side	Orthogeriatric care	Home	Nursing facility	Another medical department	In-patient mortality
Hip	55 (43.0%)	26 (20.3%)	32 (25.0%)	10 (7.8%)	5 (3.9%)
Proximal femur	82 (51.9%)	18 (11.4%)	26 (16.5%)	19 (12.0%)	13 (8.2%)
Periprosthetic proximal femur	18 (60.0%)	3 (10.0%)	5 (16.7%)	3 (10.0%)	1 (3.3%)
Proximal humerus	18 (43.9%)	18 (43.9%)	4 (9.8%)	0 (-)	1 (2.4%)
Pelvic ring	41 (44.1%)	31 (33.3%)	7 (7.6%)	10 (10.7%)	4 (4.3%)
Thoracolumbar	26 (35.1%)	26 (35.1%)	7 (9.5%)	11 (14.9%)	4 (5.4%)
Cervical	5 (16.1%)	16 (51.6%)	4 (12.9%)	2 (6.5%)	4 (12.9%)

### Outcome of orthogeriatric care

From acute trauma care 245 patients were admitted to geriatrics with weekly orthopedic consultant service for orthogeriatric care. Mean hospital stay in geriatrics was 23.4 ± 13.5 days. During the treatment 12 patients (4.9%) had surgical complications and 109 (44.5%) had non-surgical complications. Out of the 245 patients 197 lived at home and 48 lived in a nursing facility before injury. From the patients living at home 51.3% (n = 101) could return to their homes, while 26.9% (n = 53) were released to a nursing facility. Out of the remaining patients 16.2% (n = 32) were discharged to another hospital and 5.6% (n = 11) of the patients died during hospital stay. Patients living in a nursing facility before the injury showed a significantly higher mortality (5.6% vs 10.4%; p < 0.05). Logistic regression showed that the risk for transfer to a nursing home was lowered by factor 0.22 (p < 0.05) for thoracolumbar fractures and increased by factor 3.95 (p < 0.05) for periprosthetic fractures. Age increased the risk by 1.067 per year of age (p < 0.05) for discharge to a nursing facility. Discharge modality after geriatrics with orthopedic consultant services shows Tab. 4.

**Table 4:** Discharge modality out of geriatrics with orthopedic consultant depending on pre-injury living modality

Housing before injury	Home [n]	Nursing facility [n]	Anther Hospital [n]	Dead [n]	Total [n]
Home	51.3% [101]	26.9% [53]	16.2% [32]	5.6% [11]	100% [197]
Nursing facility	0.0% [0]	77.1% [37]	12.5% [6]	10.4% [5]	100% [48]

**Fig. 2:**
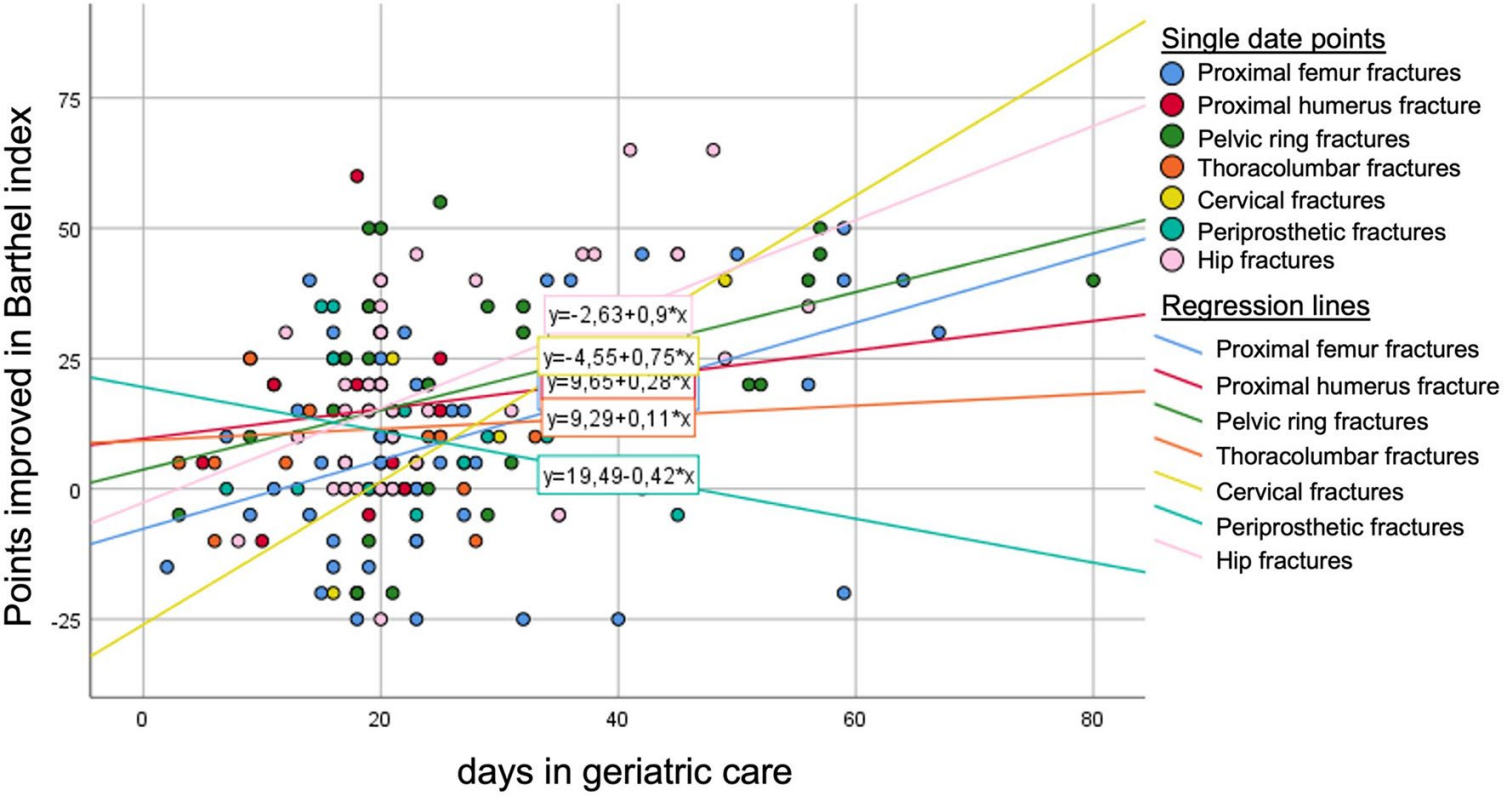
Barthel index improvement for different fracture types during orthogeriatric care. Patients with hip fractures and pelvic ring fractures improved significantly more compared to other injuries (p < 0.05)

BI could be evaluated for 191 out of 245 patients in geriatrics with orthogeriatric care. Mean BI at admission was 35.6 ± 16.0 and improved to a mean value of 51.2 ± 26.4 at discharge. Patients living at home before the injury had a significantly higher improvement compared to people living in a nursing home (20.5 ± 19.5 vs. 8.7 ± 18.0 points; p < 0.05). Logistic regression showed that the chance of BI improvement over 19 points was significantly higher by factor 4.4 for hip fractures and by factor 5.8 for pelvic ring fractures compared to the other injuries (p < 0.05). For patients living at home before injury the chance of BI improvement tended to be significant higher by factor 2.3 (p = 0.067). Improvement of the BI over time for the different injuries shows Fig. 2.

Outcome was heavily influenced by the mental state of the patients. Patients with diagnosed dementia improved significantly less during orthogeriatric care (BI improvement 6.8 ± 17.8 vs. 22.4 ± 18.3 points; p < 0.05). Compared to patients suffering from dementia mentally healthy patients had a 4.5 times higher chance of an improvement above 19 points (p < 0.05). Furthermore, patients with dementia were significantly more often released into a nursing facility (58.5% vs. 36.1%; p < 0.05) and were released earlier out of orthogeriatric care (21.9 d ± 10.0 vs. 28.8 d ± 15.4; p < 0.05).

## Discussion

Multidisciplinary orthogeriatric treatment of older hip fracture patients has multiply shown to improve patient’s outcome [8–10, 15]. Exemplarily, Prestmo et al. showed a better functional outcome [16] as well as Rapp et al. showed a reduced mortality of hip fracture patients after orthogeriatric care [17]. The aim of this work was to illuminate the outcome of different fragility fractures after orthogeriatric care.

Different orthogeriatric care models are co-existing, like geriatric ward with orthopaedic consultant service, orthopaedic ward with geriatric consultant service or integrated care models. All have shown their benefits on patient’s outcome [5, 8]. In our department we are running a two centre orthogeriatric care model. All patients ≥ 70 years with hip, proximal femur, periprosthetic, spinal, pelvic, and humerus fractures are screened in our traumatology department by ISAR score at admission. Patients with the need of orthogeriatric care (ISAR ≥ 2) receive further assessment and treatment by a geriatric consultant. More than 40% of all patients needed further care and were discharged to orthogeriatric care in geriatrics with high age, surgical treatment, non-surgical complications and high CCI as positive predictors. Patients with cervical spine fractures significant more often returned directly home compared to all other fracture types, which may underline the importance of mobility to avoid further need of care. Contrary to this, patients with cervical spine fractures showed the highest in-hospital mortality. Over 40% of the patients in our study cohort were patients with other fractures separate from fractures of the hip joint and proximal femur, which emphasizes the need for orthogeriatric care during management of other injury types as well.

Mortality after hip fractures is still devastating with 30-day-mortality of around 10% and 1-year-mortality over 20% [17–19]. In our patient cohort overall in-patient mortality was 8.6%. During further orthogeriatric care in geriatrics in-patient mortality was 6.5%. Nursing home residents were at a significantly higher risk (10.4%) during inpatient stay compared to patients living at home (5.6%). Considering, that fragility fractures of different modalities are compared to hip fractures, observed mortality is still high and emphasizes the need of orthogeriatric care not only for hip fracture patients. Similarly, Wiedl et al. observed an increased mortality after “major fragility fractures”—like pelvic, hip or thoracolumbar fractures—compared to “minor fragility fractures”—like upper extremity, cervical spine or rib fractures—in 1-year but not in 2-year follow-up [14].

During orthogeriatric care overall rate of surgical complication was lower than 5%, with clear discrepancy between different fracture types, as surgical complication rate in proximal humerus fractures reached 12.1%. Rate of non-surgical complications—like for e.g., pneumonia, delirium, heart, and kidney failure—reached 44.5%. In a meta-analysis of surgery geriatric patients, excluding cardiac surgery patients, Luger et al. reported postoperative complication rates between 6.9 and 25% [20]. The German national hip fracture database reports a non-surgical complication rate of approximately 10% after hip fractures [21, 22]. Flikweert et al. stated a long-term complication rate of 75% during 6 months after surgery, whereas half where classified as major complications [23]. Observed differences may arise from different recording periods and recording modalities but our reported non-surgical complication rate is located at the upper end compared to rates from literature [23–26], which may show that our screening successfully identified high-risk patients. In addition, it must be noted that all patients with frailty fractures are at high risk for complications, with injuries of the proximal femur as major risk factor.

More than half of patients living at home before fracture event could return to their home, while one quarter of patients were released to a nursing home. The risk for transfer to a nursing facility was lower for patients with thoracolumbar fractures (OR = 0.22) and increased multiple for periprosthetic fractures (OR = 3.95), which identifies periprosthetic fractures as a high-risk factor for need of long-term care. Observed poor outcome may be due to required limited weight bearing after surgical care and might be causal for observed poor improvement of BI in periprosthetic fracture patients.

The BI was recorded as marker for patient’s independence and need of care. Patients in orthogeriatric care showed significant improvement of BI between admission and at discharge. Patients living at home before fracture showed a significant higher improvement compared to nursing home residents (20.5 ± 19.5 vs. 8.7 ± 18.0 points; p < 0.05). Poor outcome of nursing home residents after fragility fractures is commonly known. Gosh et al. observed a 1-year-mortality rate of 29.4% in a group of long-term care residents after fragility fractures and orthogeriatric co-management. But, the author also found an BI improvement from day 5 to 3-month follow-up in nursing home residents, which indicates an existing rehabilitation potential [27]. The chance of Barthel index improvement over 19 points was significantly increased in patients with hip fractures and pelvic ring fractures. This finding may be attributed to patient’s regained mobility during orthogeriatric care.

Devastating results showed patients with diagnosed dementia. Those patients benefitted significant less from orthogeriatric care compared to mentally healthy patients, who had a 4.5 higher chance to improve Barthel index over 19 points. Our results identify dementia patients as highly vulnerable and with little recovery potential. Poor recovery progress may have resulted in observed significantly earlier discharge from orthogeriatric care of dementia patients. Future challenges consist in developing specific dementia programs to improve outcomes of this special patient group. Our findings are supported by hip fracture data, as dementia patients are identified as high-risk patients with less functional recovery potential and increased mortality [28, 29].

Data on the outcome of orthogeriatric care in the course of different fragility fractures besides hip fractures is lacking. We and few others could show that all geriatric patients are at high-risk for non-surgical complications and benefit from a standardised orthogeriatric assessment regardless of fracture modality [14, 27, 30]. Therefore, for a modern holistic orthogeriatric approach it is crucial to identify high-risk patients that require special care to improve our patient’s outcome and to enable a better use of the limited resources of healthcare systems.

Due to the retrospective design, one limitation of our study is a missing follow-up that should be part of future studies. Additionally, factors affecting patient’s outcome are multifactorial. Exemplarily, Luger et al. showed in a meta-analysis that type of anaesthesia affects morbidity and mortality of geriatric surgery patients [20]. We did not record the anaesthesia modality for the surgical patients. Therefore, further work could examine the influence of other factors, such as duration of the surgery, type of anaesthesia or bleeding management, on patient outcome.

## Conclusion

All patients with fragility fractures are at high risk for non-surgical complications and benefit from standardised orthogeriatric co-management. This work identifies several risk factors associated with a poor outcome, as dementia and periprosthetic fracture patients benefit significantly less from orthogeriatric care, while hip fracture patients benefit significantly, but showed the highest rate of transfer to a nursing home.

## Data Availability

The data that support the findings of this study are available from the corresponding author upon reasonable request, [CP].
